# Mechanism of action and therapeutic value of anoctamin-1 in gastrointestinal cancers

**DOI:** 10.3389/fimmu.2025.1599100

**Published:** 2025-05-06

**Authors:** Xiaoyue Zhang, Jie Lin, Hongpan Xu, Yan Zhou, Zhiyi Mu, Ruizhe Shi, Yalei Lv

**Affiliations:** ^1^ Oncology Department 4, The First Hospital of Hebei Medical University, Shijiazhuang, Hebei, China; ^2^ Department of Gastrointestinal Disease Center, The First Hospital of Hebei Medical University, Shijiazhuang, Hebei, China; ^3^ Department of Health Management Center, The First Hospital of Hebei Medical University, Shijiazhuang, Hebei, China

**Keywords:** anoctamin-1, gastrointestinal cancers, mechanism, therapy, prognosis

## Abstract

Gastrointestinal (GI) cancers are main causes of poor health, with most remaining difficult to treat effectively. Identifying new targets for treatment is crucial for improving the efficacy of tumour therapies and enhancing patient quality of life. Anoctamin-1 (ANO1), a crucial component of calcium-activated chloride channels (CaCCs), is expressed widely in various cell types, including epithelial cells, vascular smooth muscle cells, and tumour cells, and influences cell proliferation and migration. Nonetheless, the exact pathways through which ANO1 contributes to malignant transformation and immune responses remain elusive. This review comprehensively examines the regulatory functions and potential therapeutic applications of ANO1 in GI cancers. The goal of this work is to offer new perspectives for further study on the role of ANO1 in gastrointestinal cancers and to support improvements in therapeutic strategies for cancer diagnosis and treatment through the targeting of ANO1.

## 1 Introduction

The treatment of gastrointestinal (GI) cancers has long been significant subjects in medical research. Important progress has been made in treating GI cancers through advances in medical technology and research. However, the complex and variable pathogenesis of tumours creates challenges such as recurrence, metastasis, and drug resistance, which pose major threats to human health (1, 2). Data from the International Agency for Research on Cancer revealed that the global incidence of cancer was approximately 20 million cases, with 9.7 million fatalities in 2022. Colorectal cancer is the most common and the most fatal GI cancer, accounting for 9.6% of all cancers and 9.3% of all cancer-related deaths, followed by cancers of the stomach, liver, oesophagus, and pancreas (3).

The treatment of GI cancers typically involves surgery, chemotherapy, radiotherapy, targeted therapy, and immunotherapy (4–6). However, patients may respond differently to treatments due to tumour heterogeneity. Moreover, adverse reactions such as resistance to chemotherapy and systemic toxicity may occur as tumours develop (7). Compared with traditional chemotherapy, targeted therapy offers advantages such as high specificity, efficacy, and low toxicity. Over the past 30 years, targeted therapy has proven effective for treating various types of cancer, achieving significant results. However, challenges such as a limited number of targets, the occurrence of off-target toxicity, and development of resistance have hindered the clinical application of targeted therapies (8, 9). Thus, there is a pressing need to increase overall survival or quality of life through discovery of novel targets and therapeutic approaches. Ion channels such as transient receptor potential channels, volume-regulated anion channels, and calcium-activated chloride channels (CaCCs) are integral to various pathophysiological activities are abnormally expressed in GI cancers. Among these, CaCCs have garnered significant attention due to their critical functions in cancer progression (10–12).

Anoctamin-1 (ANO1), also known as transmembrane protein 16A (TMEM16A) or discovered on gastrointestinal stromal tumour 1 (DOG1), is a member of the CaCC family. The gene that encodes ANO1 is located on chromosome 11q13. Initially discovered in Xenopus oocytes, ANO1 is localized primarily on the plasma membrane (13). As an essential component of voltage-dependent channels, ANO1 activates chloride ion transport through intracellular calcium ions, thereby regulating the cell’s membrane potential, calcium balance, and excitability (14, 15). It is involved in various biological processes that are linked to malignancies, including mucus secretion, neuronal excitability, cell proliferation, and signal transduction (16–19). ANO1 plays a key role in gastrointestinal cancers, and targeting the pathway through which it acts may be an effective treatment strategy.

Interventions that inhibit ANO1 or related pathways can reduce its activity and thereby suppress tumorigenesis and progression. Most currently known ANO1 modulators are channel inhibitors that affect chloride and calcium channel currents in a concentration-dependent manner (20–22). Some ANO1 inhibitors block ion conduction pores, induce pore closure, and inhibit channel activity (23). Other inhibitors downregulate ANO1 expression; in addition, targeting the ion-binding sites of ANO1 is a feasible approach. Preclinical studies have confirmed the efficacy of ANO1 inhibitors (24, 25). However, additional clinical trials are needed to verify their functions.

This review describes the role of ANO1 and its regulatory mechanisms in gastrointestinal cancers and the results of ANO1 inhibitors *in vivo* and *in vitro* studies. ANO1 expression is upregulated in various GI cancers, involved in tumour invasion, metastasis, or drug resistance, and associated to poor prognosis in patients with these cancers. The mechanisms by which ANO1 promotes the malignant behaviour of tumour cells are complex and primarily include the regulation of upstream noncoding RNAs and the activation of multiple downstream signalling pathways. The tumorigenic effects of ANO1 can be inhibited by blocking channel current conduction, reducing channel activity, or decreasing ANO1 expression. ANO1 may serve as a therapeutic target and novel prognostic biomarker. Further exploration of the role of ANO1 in gastrointestinal cancer is crucial for advancing tumour diagnosis and treatment.

## 2 Structure and physiological function of ANO1 in gastrointestinal cells

The anoctamin family includes 10 members, anoctamin 1–10, all of which exhibit high sequence conservation. Among them, ANO1 and ANO2 are CaCCs (26), whereas ANO3–7 and ANO9 function as Ca^2+^-dependent lipid scramblases (27–32). ANO8–10 are are mostly retained in the cytoplasm (33–35). ANO1 displays characteristics typical of CaCCs, including Ca^2+^ and voltage-dependent activation and anion selectivity. Notably, the sequence of ANO1 contains at least four alternatively spliced exons (designated a, b, c, and d), producing proteins of 712 to 1006 amino acids (36, 37).

**Figure 1:**
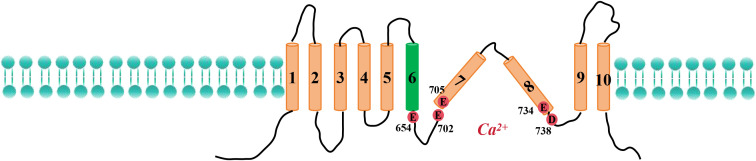
Schematic representation of the structure of ANO1.

Paulino et al. used cryoelectron microscopy to determine that the ANO1 protein forms a homodimer, each subunit of which contains two Ca^2+^-binding sites and 10 transmembrane helices (38). The extracellular region of the protein contains cyclic folded domains that are formed by amino acid residues that link α1–α2, α5–α6, and α9–α10. Both the N-terminus and the C-terminus of ANO1 reside on the cytoplasmic side of the membrane. Research has revealed that the Ca^2+^ binding site on ANO1 contains five negatively charged residues—E654, E702, E705, E734, and D738—that coordinate the binding of two calcium ions. These residues are distributed across transmembrane helices α6–α8 (**Figure 1**). In addition, mutations of N650, N651, and N730 to alanine have been shown to reduce the affinity of ANO1 for Ca^2+^, suggesting that these residues also contribute to calcium ion binding (39–41).

The subunit cavity within the ANO1 structure forms an ion conduction pore that mediates ion permeation. Transmembrane helices α3–α7 surround this pore, forming a closed channel resembling an hourglass. Electrophysiological experiments and mutagenesis have shown that R515 (in α3) and K603 (in α5–α6) are key to anion selectivity. Further studies indicated that seven residues within the pore region contribute to the calcium-dependent gating mechanism of ANO1. Specific mutations, such as N546A, I550A, Y593A, I596A, and F712A, increase the apparent affinity of ANO1 for calcium, whereas the V59A and L643A mutations decrease it (42).

The fact that ANO1 is expressed in various gastrointestinal tissues highlights its involvement in several physiological roles. ANO1 is highly expressed on membranes of the interstitial cells of Cajal, where ANO1 maintains cell excitability by regulating generation and propagation of slow-wave currents and contributes to the secretion of digestive fluid and intestinal peristalsis (43). ANO1 is present at the apical membranes of colonic and jejunal epithelial cells and contributes to Cl^-^ efflux. Inhibition of ANO1 causes mild oedema in the intestine, suggesting that blocking ANO1 may be an effective method of treating enterotoxin-related diarrhoea (44). A reduction in the amount of ANO1 in gastric muscles leads to the loss of gastric slow waves and an irregular spike complex, resulting in increased gastric emptying time (45). ANO1 can also activate bile duct epithelial cells through the ATP-Ca²^+^-PKCα pathway and thereby increase bile secretion (46). In pancreatic islet β cells, ANO1 interacts with the INS promoter to regulate electrical activity and promote insulin secretion (47). ANO1 acts as an HCO_3_^-^ transport channel In pancreatic acinar cells, regulating the luminal pH and providing a therapeutic approach for acute pancreatitis (48). In lacrimal and salivary gland acinar cells, ANO1 interacts with transient receptor potential vanilloid 4 (TRPV4), inducing cell contraction by increasing intracellular Ca²^+^-induced chloride currents and thereby promoting saliva and tear secretion (49). ANO1 activation promotes the proliferation of smooth muscle cells in portal vein and contributes to liver fibrosis and portal vein wall thickness, two characteristics associated with portal hypertension and chronic liver disease (50, 51).

## 3 Research on ANO1 in gastrointestinal cancers

### 3.1 Research on ANO1 in colorectal cancer

CRC ranks third among malignant tumours in terms of occurrence. More than 2.2 million new cases and 1.1 million deaths due to CRC are projected to occur by 2030, representing a 60% increase in the worldwide burden of this disease (52, 53). ANO1 has been identified as a novel oncogene in CRC (54, 55). According to The Cancer Genome Atlas database, ANO1 expression is upregulated in CRC, and associated with metastasis and immune regulation. It is also significantly related to higher tumour-node-metastasis (TNM) stage and worse prognosis (56, 57).

Inhibition of ANO1 can downregulate the expression of the membrane proteins frizzled protein 1 (FZD1) and β-catenin, increase the level of glycogen synthase kinase-3β (GSK3β), disrupt the Wnt/β-catenin signalling pathway, and reduce the proliferative capacity of CRC cells (54).

Yuen et al. analysed two independent CRC patient datasets from the Gene Expression Omnibus and reported that high expression of the transcription coactivator PDZ-binding motif (TAZ), its downstream targets AXL and connective tissue growth factor (CTGF) is associated with shortened survival. Further analysis of patients with co-overexpression of TAZ–AXL–CTGF revealed lower ANO1 levels are significantly associated with better survival. Combined analysis of ANO1 and TAZ–AXL–CTGF expression levels can accurately predict prognosis of patients (58).

The occurrence or development of CRC is regulated by ANO1 through interactions with microRNAs. Upregulated ANO1 expression in CRC is accompanied by downregulated expression of tumour-suppressing microRNAs including miR-144, miR-132, miR-9, and miR-18a. MiRNAs can integrate with the 3’-untranslated region of ANO1 mRNA, downregulate its expression, and reduce the invasion of CRC cells. MiR-9 can limit the invasive capacity of CRC cells by directly inhibiting ANO1. This is achieved by decreasing the expression of downstream molecules such as Ser and Thr kinase (AKT), cyclin D1, and extracellular signal-regulated kinase (ERK) (59). Overexpression of miR-144 can target ANO1 and suppress the epidermal growth factor receptor (EGFR)/ERK signalling pathway. Patients with colorectal cancer whose miR-132 expression is low and whose ANO1 expression is high have shorter progression-free survival (60, 61). MiR-18a regulated *F.nucleatum-*mediated chemoresistance in colorectal cancer, but the specific mechanism remains unclear (62). Another study reported the gut microbiome can increase ANO1 expression by co-culture *F.nucleatum* with colon cancer cell lines HCT116 and HT29, thereby reducing apoptosis induced by oxaliplatin and 5-fluorouracil in CRC cells. Consistently, ANO1 silencing could reverse *F.nucleatum* effects and increase apoptosis. The results demonstrated that *F.nucleatum* promoting resistance to chemotherapy via ANO1 pathway (63). These studies suggest that the miRNA/ANO1 axis is crucial in CRC and that it represents a potential therapeutic target.

### 3.2 Research on ANO1 in gastric cancer

Epithelial–mesenchymal transition (EMT), lymph node metastasis, and poor prognosis are clinicopathological characteristics associated with ANO1 overexpression in gastric cancer tissues (64–66). MiR-381 functions as a tumour suppressor by targeting ANO1. It inhibits invasion and metastasis of cancer cells by inactivating transforming growth factor-β (TGF-β) signalling; which in turn decreases EMT-related genes such as vimentin, fibronectin, and N-cadherin (67). The ANO1 promoter was shown to be activated by signal transducer and activator of transcription 6 (STAT6). Inhibition of the STAT6/ANO1 pathway reduced proliferation, migration, and invasion by gastric cancer cells, suggesting that the STAT6/ANO1 pathway could represent a novel therapeutic target for gastric cancer.

### 3.3 Research on ANO1 in liver cancer

Most cases of primary liver cancer are hepatocellular carcinoma (HCC), a cancer that now ranks sixth globally. In 2022, more than 860,000 new cases of liver cancer were reported, accounting for approximately 4.3% of all malignancies; in addition, 7.8% of all cancer deaths (more than 750,000) were attributable to liver cancers (2, 3). Most patients are diagnosed with liver cancer only after it has reached advanced stages, which leads to the poor prognosis of this disease. The statistics indicate that current treatments for liver cancer are insufficient, and this has prompted investigations into tumour mechanisms so that new therapeutic approaches can be developed. One potential target for such research is ANO1.

Analysis of genomic data from primary HCC revealed the expression of ANO1 mRNA and protein is noticeably greater in HCC tissues than in adjacent noncancerous tissues. Distant metastasis is positively associated with high ANO1 expression (68). ANO1 is essential for tumour maintenance and is considered a potential driver of HCC. Overexpression of ANO1 is related to poor survival and vascular invasion, suggesting that ANO1 is a high-risk marker for HCC (69). Overexpression of ANO1 induced the upregulation of phosphatidylinositol 3-kinase (PI3K), phosphorylated AKT (pAKT), phosphorylated p38 (p-p38) and phosphorylated ERK (pERK) in HepG2 and SMMC7721 cells. The results suggested ANO1 promoted the proliferation of HCC cells by inducing the expression of PI3K/AKT and the mitogen-activated protein kinase (MAPK) signaling pathway. ANO1 regulates the cell cycle, increasing the number of S-phase cells and decreasing the number of G-phase cells. Inhibition of ANO1 results in decreased cell viability and increased apoptosis *in vitro* and *in vivo (* 70). Deng et al. also reported ANO1 siRNA suppressed proliferation, invasion, and migration by SMMC-7721 cells, which accompanied inhibited MAPK signalling pathway by phosphorylation of ERK1/2 and p38 reduction and cyclin D1 induction (71). This evidence indicates that ANO1 induces carcinogenesis and it represents an effective therapeutic target for HCC.

### 3.4 Research on ANO1 in oesophageal squamous cell carcinoma

Oesophageal cancer ranks seventh as a cause of mortality and eleventh in incidence, making it a major public health concern worldwide. According to reports, ESCC is a common subtype of oesophageal cancer in China. Among all malignant tumours in China, oesophageal cancer ranks 5th in mortality and 6th in incidence (72, 73). Common risk factors for ESCC include smoking, excessive alcohol consumption, dietary influences, genetic predispositions, microorganisms, and other environmental factors (74, 75).

Deng et al. reported increased ANO1 levels in ESCC tumours and the corresponding lymph nodes as well as in metastatic tumours (76). Kaplan-Meier survival analysis revealed patients with positive ANO1 expression had poorer prognoses than did those with negative ANO1 expression; the two groups had overall survival rates of 26.22% vs. 42.91%, respectively (77).

When ANO1 binds to JUN, the liver X receptor (LXR) pathway is rendered inactive, and intracellular cholesterol buildup is increased; this in turn inhibits transcription of the genes that encode cholesterol hydroxylase, cytochrome P450 (CYP) enzymes, and CYP27A1. Additionally, in ESCC cells with high ANO1 expression, the inhibitory effect of the LXR pathway on interleukin-1β (IL-1β) is reduced, leading to increased secretion of IL-1β, activation of nuclear factor-kappa B (NF-κB) signalling in fibroblasts, and production of chemokine CCL1, thereby increasing the invasiveness of ESCC cells. Consequently, increased ANO1 levels in cancer cells trigger both intracellular and extracellular pathways that modify cholesterol metabolism and activate fibroblasts, thus facilitating cancer metastasis (76).

In oesophageal cancer, the long noncoding RNA (lncRNA) GIHCG is significantly expressed. GIHCG inhibits miR-29b-3p, and this leads to increased ANO1 expression and promotes proliferation and invasion by oesophageal cancer cells. GIHCG knockdown suppresses tumour growth by reducing the direct binding of GIHCG to miR-29b-3p and inhibiting ANO1 production. This finding revealed that the lncRNA GIHCG/miR-29b-3p/ANO1 molecular axis plays an important role in ESCC (78).

### 3.5 Research on ANO1 in pancreatic cancer

Tumour recurrence and metastasis pose significant obstacles to effective treatment of pancreatic cancer (79, 80). Sixty-one percent of pancreatic cancer tissues have high levels of ANO1 expression, and patients with ANO1-positive tumours have worse overall survival than patients who do not (81, 82). In AsPC-1 cells, absence of ANO1 inhibits the phosphorylation of EGFR and AKT and induces apoptosis (83). Overexpression of ANO1 and oncogenic KRAS in cancer cells were demonstrated to increase cell proliferation *in vivo* and vitro. RNA-seq analysis were performed in cancer cells bearing different status of ANO1 and KRAS. The results revealed that high levels of ANO1 and KRAS were associated with activating key genes involved in lipid metabolism like HMGCS1, indicating that ANO1 regulated basic metabolic processes that occured in pancreatic cancer cells. However, the specific mechanism through which this occurs requires further research (84).

### 3.6 Research on ANO1 in gastrointestinal stromal tumours

GIST, which originates from Cajal cells, is the most prevalent sarcoma of the gastrointestinal system (85). GISTs consist primarily of spindle or epithelioid cells. Genetically, they often feature mutations in platelet-derived growth factor receptor alpha (PDGFRα) or c-kit. Immunophenotypically, 95% of GISTs are positive for CD117 (86). ANO1 was initially identified in GISTs and is highly expressed in 65–100% of cases. It is now included alongside CD117 in the diagnosis of GISTs and serves as a specific diagnostic marker (87, 88).

According to Miettinen et al., Cajal and gastric epithelial cells test positive for ANO1. Overall, the sensitivities of patients with GISTs to ANO1 and KIT are very similar, at 94.4% and 94.7%, respectively. The expression of ANO1 is more than that of KIT in GISTs with PDGFRα mutations (89). ANO1 expression was assessed in 59 GIST patients by Rizzo et al., who reported its expression was significantly related to tumour size and that it was present in 66% of CD117-positive GISTs. Patients with high ANO1 expression exhibit worse prognosis at 2 years after treatment (66% vs. 100% recurrence-free survival) (90). Knockdown of ANO1 in the GIST xenograft model dramatically reduced tumour development. According to ingenuity pathway analysis, ANO1 modulates the antiangiogenic factor insulin-like growth factor-binding protein 5 (IGFBP5), which in turn regulates IGF/IGFR signalling in the tumour microenvironment and hence promotes tumour growth (91). In flow cytometry experiments, GIST cells were incubated with ANO1 inhibitors. The results showed inhibition of ANO1 may shift early apoptotic cells to late apoptotic stages (92). Another study reported biochemical inhibition of ANO1 reduced cell viability and lead to G_1_ cell cycle arrest, indicative of apoptosis (93). These studies indicate ANO1 may serve as a biomarker for GIST and is positively correlated with poor prognosis, making it a specific target for therapy.

## 4 Targeting ANO1 in gastrointestinal cancers

Due to its significant functions in cancer, ANO1 is recognized as a potential clinical therapeutic target. Various ANO1 inhibitors have been identified through high-throughput screening in which iodine-sensitive yellow fluorescent protein (YFP) was employed (94–98). CaCCinh-A01 is the most commonly used ANO1 inhibitor (98–101). Site-directed mutagenesis experiments have shown that CaCCinh-A01 binds to the R515/K603/E623 sites of ANO1, blocking the ion conduction pore and significantly inhibiting ANO1 chloride ion currents while promoting protein degradation (102). CaCCinh-A01 has demonstrated antitumour effects in pancreatic ductal adenocarcinoma (PDAC), CRC, breast and prostate cancer, head and neck squamous cell carcinoma (HNSCC), and glioblastoma (GBM) (103–108). It reduced ANO1 protein expression by promoting proteasomal turnover of ANO1 in parental cancer cells. In CaCCinh-A01-resistant cell pools, it failed to decrease ANO1 protein levels with inhibited ANO1-dependent currents. Knockdown of ANO1 in CaCCinh-A01-resistant cell pools led to significantly decrease colony formation. The results showed CaCCinh-A01 may play its antitumor role by decreasing ANO1 protein levels rather than inhibiting ANO1 channel activity (104). Apart from tumor cells, it also reduces allodynia in nerve injury (109). CaCCinh-A01 acts as a Cl^-^ channel inhibitor to promote cells proliferation and wound healing ability of airway (110). CaCCinh-A01 effectively prevents the progression of kidney fibrosis by inhibiting ANO1 expression (111). T16Ainh-A01, an effective inhibitor of ANO1, effectively blocks chloride ion currents with an IC50 of less than 1 μmol. T16Ainh-A01 significantly inhibits cell migration, induces cell cycle arrest, and promotes apoptosis of prostate and colon cancer cells (107); its antitumour effects have also been observed in PDAC, HNSCC, and breast cancer (112–114). The small molecular compound K786-4469 binds to ANO1 at Arg 557, reducing ANO1 expression and reversing disordered cholesterol metabolism, thereby restoring cholesterol homeostasis. In ESCC cells and mouse models, K786-4469 significantly decreases the expression of ANO1 and its downstream target CYP27A1 and reduces intracellular cholesterol levels, thereby mitigating lung metastasis of ESCC (76). In prostate cancer, breast cancer, and PDAC, Ani9 inhibits ANO1 synthesis in a concentration-dependent manner, reducing the proliferation and invasive capabilities of cancer cells (115, 116).

Natural products obtained from various plants have been found to inhibit ANO1. In CRC, dehydroandrographolide (DP) can significantly inhibit chloride ion currents in SW620 cells, reduce ANO1 protein expression, and suppress the activity and migration of tumour cells (117). Honokiol binds to ANO1 at R429/K430/N435, blocking its channel currents and inhibiting the proliferation of CRC cells (118). Several drugs in clinical use have been shown to inhibit ANO1. Idebenone, an analogue of coenzyme Q10, has demonstrated clinical effects in treating Alzheimer’s disease (119). It inhibits ANO1 channel activity and has antitumour effects on prostate and pancreatic cancer cells in a dose-dependent manner (120). Jiang et al. evaluated benzbromarone, a drug used to treat gout, in GI cancer cells and patient-derived xenograft (PDX) models. It is also reported benzbromarone strongly inhibited ANO1 protein expression in gastric, oesophageal, and CRC cells and exerted antitumour effects in gastric cancer and GIST PDX models (9). Evodiamine and rutecarpine, compounds extracted from the traditional Chinese medicine *Evodia rutaecarpa*, bind to Lys^384^, Thr^385^, and Met^524^ in ANO1, inhibiting Cl^-^ currents and suppressing peristalsis in isolated guinea pig ileum (121). Diltiazem, a calcium channel blocking agent that is used to treat cardiovascular disorders, can significantly inhibit invasion by HCC cells by downregulating the expression of ANO1 (122). **Table 1** lists some chemicals that have been shown to inhibit ANO1.

**Table 1:** Summary of chemicals used to inhibit ANO1.

Chemicals	Molecular Mechanism	Effects	Cancer Types	References
CaCCinh-A01	binds to R515/K603/E623 and blocks the pore of ANO1 inhibits Cl^-^ efflux promotes degradation of the ANO1 protein	inhibits cell proliferation, invasion and migration	PDAC, HNSCC, GBM, CRC, breast and prostate cancer	(103–108)
T16Ainh-A01	inhibits Cl^-^ efflux	inhibits cell proliferation and migration	PDAC, HNSCC, CRC, breast and prostate cancer	(107, 112–114)
K786-4469	binds to ARG557 in ANO1	decreases cholesterol levels, inhibits cell invasion and metastasis	ESCC	(76)
Ani9	decreases ANO1 protein levels	decreases cell viability and proliferation	HNSCC, PDAC, breast and prostate cancer	(115, 116)
Dehydroand- rographolide	inhibits chloride currents decreases protein levels of ANO1	inhibits cell proliferation and migration	CRC	(117)
Honokiol	binds to R429/K430/N435 of ANO1 inhibits Cl^-^ currents	inhibits cell proliferation	CRC	(118)
Idebenone	inhibits Cl- efflux	decreases cell proliferation and migration	PDAC prostate cancer	(120)
Benzbromarone	decreases levels of ANO1 protein	decreases cell proliferation inhibits tumour growth in PDX model	ESCC, CRC, GC, GIST	(9)
Evodiamine and rutecarpine	bind to Lys^384^, Thr^385^, and Met^524^ inhibit Cl^-^ currents	suppress peristalsis in isolated guinea -pig ileum	---	(121)
Diltiazem	decreases mRNA and protein levels of ANO1	inhibits cell invasion	HCC	(122)

## 5 Conclusion

GI cancers of the colorectum, stomach, liver, oesophagus, pancreas and mesenchyme are prevalent cancers with high mortality and morbidity. Risk factors for GI cancers include obesity, alcohol consumption, smoking, inflammatory diseases, viral infections and mutations. Routine endoscopy is recommended for screening neoplasms. However, due to the limited availability of endoscopy in some high-risk regions, many patients are diagnosed with disease at an advanced stage; in addition, the incidence of GI cancers among young adults is increasing. The treatment of GI cancers is complex and may include a combination of surgery, chemotherapy, radiation therapy, immunotherapy and targeted therapy. However, these interventions can have numerous side effects, including systemic toxicity, the development of resistance, and a need for supportive care. Moreover, treatment efficacy may be restricted by metastasis and recurrence. Therefore, elucidating the mechanisms involved in tumour progression and identifying novel therapeutic targets are urgent objectives in GI cancer research.

The progression of GI cancers is influenced by various factors, including gene editing, tumour size and location, microenvironmental regulation and host susceptibility. An increasing number of studies have characterized ANO1 as a chloride channel whose molecular expression significantly impacts cancer at various stages. Its effects are transduced through a variety of signalling pathways, including the TGF-β, TAZ–AXL–CTGF, PI3K/AKT, and Wnt/β-catenin pathways. Given these observations, ANO1 may be considered a potential diagnostic, therapeutic, and prognostic marker for GI cancers. Certain miRNAs play vital roles in the invasiveness and migration of GI cancers. These genes are negatively correlated with ANO1 and are linked to vascular invasion or lymph node metastasis. In other cancers, such as HNSCC and breast cancer, ANO1 is also regulated by other factors, including DNA methylation and acetylation, lncRNAs, and circular RNAs (circRNAs) (10, 123–125). ANO1 is also vital in modulating the tumour microenvironment through its effects on cell cycle or host’s immune response to tumours. Due to these key roles, ANO1 holds great potential as a new target for the diagnosis and therapy of GI cancers.

Current projections for ANO1-based therapies include two strategies: suppression of chloride channel activity and reduction of ANO1 expression at the mRNA and protein levels. Although ANO1 expression is upregulated in GI cancers, inhibiting the activity of ANO1 can correct the abnormal overexpression of downstream molecules. A variety of inhibitors have been identified using high-throughput screening, a technique that possesses the great advantages of high specificity and wide application. Natural inhibitors represent excellent prospects with low toxicity, good drug potential, and almost no side effects. Clinically used drugs inhibit cancer cells by downregulating ANO1, with promising safety and economic efficacy. Moreover, nonspecific inhibitors of CaCCs, including broad-spectrum inhibitors such as niflumic acid (NFA), DIDS and 4,4’-diisothiocyano-2,2’-stilbenedisulfonic acid (NPPB), are available. NFA and DIDS block voltage-gated K^+^ channels and volume-regulated anion channels as well as CaCCs (126–130), and NPPB inhibits both ANO1 and ANO2 (131, 132). The selectivity of these drugs is low, and their effects on tumour cells are not ideal; however, they have therapeutic effects on other conditions such as cystic fibrosis, hypertension, and asthma (133–137). Further investigations are needed to understand the specific mechanisms involved.

Despite the considerable therapeutic potential of ANO1, several challenges remain to be addressed. First, current research on ANO1 inhibitors is largely being conducted in preclinical stage without any inhibitors verified for clinical use. There is a need to develop animal models that allow comprehensive studies and to translate the efficacy observed in animal studies to human trials. Furthermore, the immune response induced by ANO1 inhibitors may pose a problem with respect to the safety and efficacy of therapy. Many ANO1 inhibitors such as CaCCinh-A01,T16Ainh-A01, K786-4469, Ani9, dehydroandrographolide and Idebenone are discovered from chemical and natural products. Of those, chemical inhibitors have half maximal inhibitory concentration values range from 100nM to 3µM with natural products up to 10µM or more (138–141). Determination of the appropriate dosage is equally significant, as overuse or insufficient use could impair the therapeutic effect, leading to unsatisfactory treatment outcomes. Finally, it is still remained to verify whether the inhibitors can act on other channels. A variety of ANO1 inhibitors have exhibited inhibitory effects on other ion channels such as ANO2, BEST1 and CFTR (142–144). Furthermore, ANO1 is widely expressed in no more than one tissue, including smooth muscle cells, epithelial cells and neuron. It is necessary to validate targets for selective modulators. There are various limitations related to the application of ANO1 inhibitors, understanding the mechanism of interaction of ANO1 with other elements, and further exploration of ANO1 expression levels. Addressing these limitations can improve research on ANO1-based therapies.

It is anticipated that integrating ANO1 in combination cancer therapy is a promising direction to improve therapeutic efficacy. Combined blockade of ANO1 and EGFR remarkably improved response to cetuximab in HNSCC and breast cancer (145, 146). In addition, recent evidence indicated ANO1 blockade can enhance the anti-tumor effect of cisplatin in HNSCC and lung cancer (147, 148). The resistance to Trastuzumab can be overcome by treatment with ANO1 inhibitor in mice with prostatic and breast cancer (149).

ANO1 also plays an important role in a variety of tumor infiltrating immune cells and regulating anti-tumor immune response in tumor microenvironment. Aberrant overexpression of ANO1 contributed to tumor-induced immunosuppression. In tumor cells, activated ANO1 promoted TGF-β production and release, which increased cancer-associated fibroblasts (CAFs) infiltration and inhibited accumulation of CD8^+^T cells, leading to immunotherapeutic resistance to anti-PD-1 antibody in GI cancers (9). According to data from Pan-cancer RNA-seq, immune cells such as resting CD4^+^T cells and activated mast cells were higher in ANO1-high group, while activated CD4^+^ Tcells and dendritic cells, plasma cells were higher in ANO1-low group. The results showed ANO1 may function an inhibited role in helper T cells (57). GSEA analysis of immune cells in high and low ANO1 expression group in HNSCC showed increased infiltration of naive B cells, lymphocytes and CD8 cells in low group and increased infiltration of M2 macrophages in high group (150). Functional enrichment analysis of HCC identified high expression of ANO1, CDK1 and PDGFRA predicted high-risk group for HCC. Furthermore, some immune checkpoints such as TIGT, CTLA4, LAG3 and HAVCR2 were demonstrated higher in the high-risk group (68). Wang et al. analyzed genomic data from 30 PD-L1-negative NSCLC patients who received dual immunotherapy with anti-PD-L1 and CTLA-4. Patients with ANO1 mutations who received combined therapy demonstrated longer progression-free survival, indicating that ANO1 may serve as an effective biomarker for predicting the efficacy of dual blockade therapy in NSCLC with PD-(L)1/CTLA-4 (151).

**Figure 2:**
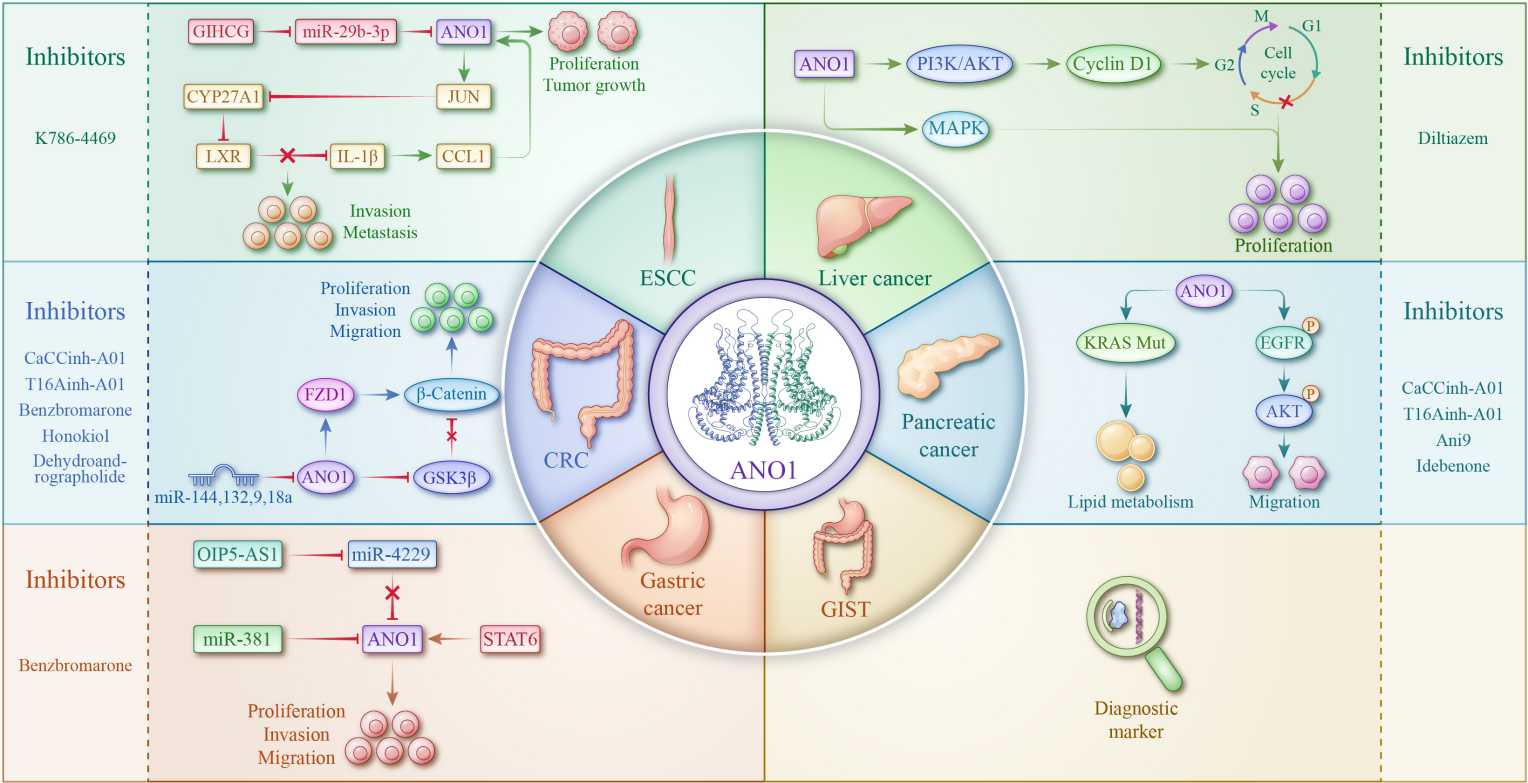
Role of ANO1 and targeting strategies in CRC, ESCC, liver cancer, gastric cancer, pancreatic cancer, and GIST.

As research progresses, the molecular mechanisms by which ANO1 mediates tumour malignancy are becoming clearer (**Figure 2**). Knowledge of these mechanisms provides a foundation for the use of ANO1 as a target for improving clinical diagnosis, precisely predicting prognosis, and accelerating the treatment of GI cancers. With the evolution of technology and research, the role of ANO1 in cancer is likely to be profound and promising.
